# Effect of Wave Attenuation on Shear Wave Velocity Determination Using Bender Element Tests

**DOI:** 10.3390/s22031263

**Published:** 2022-02-07

**Authors:** Yanbin Gao, Xiaojun Zheng, Hao Wang, Wenkang Luo

**Affiliations:** 1 Department of Geotechnical Engineering, Tongji University, Shanghai 200092, China; yanbin_gao@tongji.edu.cn (Y.G.); 1932639@tongji.edu.cn (X.Z.); 2032242@tongji.edu.cn (W.L.); 2 Institute of Acoustics, Tongji University, Shanghai 200092, China

**Keywords:** bender elements, signal interpretation, wave attenuation, shear wave, time domain method

## Abstract

Wave attenuation is a widespread physical phenomenon in most acoustic tests, but there is a scarcity of quantitative investigations into the influence of wave attenuation on the determination of shear wave travel time in bender element tests. To ascertain this attenuation effect, a series of bender element tests were conducted on clay samples with different lengths under unconfined conditions. The experimental results suggest that the real first peak of the received signal attenuates gradually with the increase of the sample length and even becomes undistinguished when the sample length exceeds a limit. This phenomenon results in misinterpretation of the wave travel time using the time domain method. In this study, the shear wave travel time is misinterpreted when wave travel distance over approximately 80 mm, leading to underestimation of the *V*_S_ by 17% for the peak-to-peak approach and 10% for the arrival-to-arrival method. Therefore, besides the near field effect and boundary reflection, the wave attenuation effect turned out to be an important factor influencing the determination of *V*_S_ using the time domain method. Accordingly, it is advisable to predetermine the limit test distance for a specific testing system under conditions, particularly for long distance testing.

## 1. Introduction

**Figure 1:**
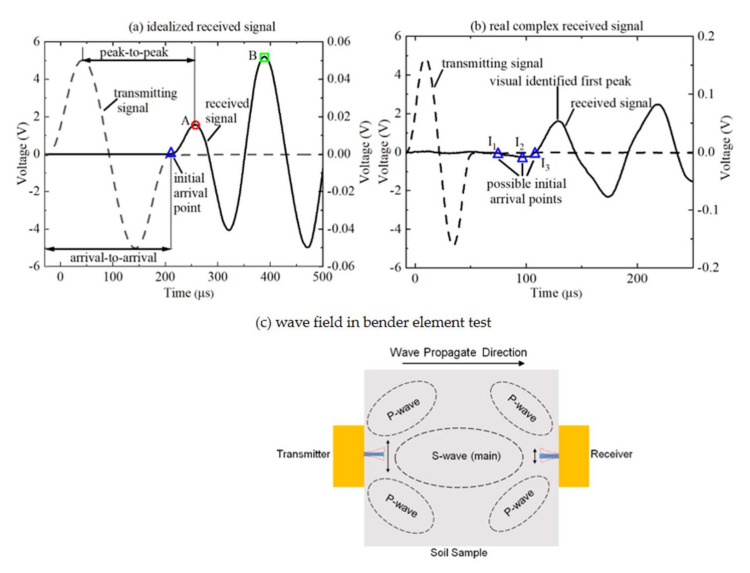
Time domain methods illustrated by (**a**) idealized received waveform, (**b**) real complex received waveform, and (**c**) wave field in bender element test.

The shear wave velocity (*V*_S_) is a fundamental soil parameter, which can be used for liquefaction evaluation [1,2], sample quality assessment [3], and small-strain shear stiffness determination [4]. Since the bender element method was first introduced for soil testing by Shirley and Hampton [5] and Shirley [6], it has been widely installed in geotechnical testing devices to obtain *V*_S_ of geotechnical materials (such as clay and sand), including oedometers, direct shear apparatuses, triaxial apparatuses, resonant columns, and even applied in scaled physical model tests. Bender element test commonly adopts transmitter–receiver measurement mode (not the resonant mode) based on the direct-arrive wave principle. The determination of *V*_S_ can be expressed as

(1) $$V_{s}=\frac{L_{tt}}{t}$$

where *L*_tt_ is the test distance or the travel distance of shear wave (S-wave), i.e., the tip-to-tip length between the bender elements of the transmitter-receiver; and *t* is the travel time of S-wave. Figure 1a shows the time domain method to determinate the travel time *t* from the transmitting and received signals, which includes the arrival-to-arrival approach and peak-to-peak approach. In fact, the bender element acts as a finite-length line source, the wave field in bender element test is complex, neither an ideal spherical wave, nor an ideal flat wave. Bender elements generate two P-wave side lobes normal to their plane one in compression and the other in rarefaction, and an S-wave frontal lobe as shown in Figure 1c. The P-wave distortion usually resulting in a reverse deflection before the arrival of the true S-wave (see Figure 1b). Thus, the initial arrival point of the S-wave is difficult to distinguish and the visual inspected first peak of the S-wave (marked by point A in Figure 1b) is also arguable [7,8,9,10,11,12,13]. This distortion is referred to as ‘near field effect’ and it tends to appear when test distance *L*_tt_ falls in the range of 0.25 to 4 wavelengths (λ) of the received wave [14,15]. In this case, it is widely accepted that point I_3_ (zero after the first bump) shown in Figure 1b approximately denotes the arrival of the S-wave [7,15,16]. The near field effect can be effectively eliminated by increase the ratio of *L*_tt_/λ, i.e., increasing the excitation frequency or the sample length [14,15,16,17,18,19,20,21,22,23]. However, for long soil sample testing, P-wave reflection from the lateral boundary of the soil sample is another potential influencing factor. It can also result in the distortion of the received signals similar with that as shown in Figure 1b. In this case, there is no reliable approach to distinguish the arrival of S-wave from the distorted received signals [24,25]. In order to avoid this kind of distortion, the ratio of length (*L*) to diameter (*D*) of the soil sample must be properly considered. Because of these complexities, no standard method has been developed to date for testing and interpretation of the test results. Hence, the results obtained from bender/extender tests are highly subjective in nature and involves high degree of uncertainty [15].

Wave attenuation is a basic physical phenomenon in most acoustic tests including bender element tests. Wave attenuation is known to be strongly related to the wave travel distance and the properties of frequency. Table 1 shows some bender element testing conducted on long soil samples or long test distance in the model box. However, literature review shows that the influence of wave attenuation on the received signals and the determination of the travel time of S-wave were rarely investigated in bender element testing. Is the wave attenuation possibly one of the sources of the uncertainty of the interpretation of the test results in bender element test? By comparing the received waveforms of three clay samples with different lengths, Brignoli et al. (1996) reported that the first peak of the received signal tends to attenuate more rapidly than the remaining part of the receiving signal as the test distance increases [26]. Therefore, when the first peak (denoted by A in Figure 1a) attenuates to an imperceptible extent, a risk will be posed because the second peak (denoted by B in Figure 1a) can easily be mistaken for the first peak. In this case, the corresponding results of calculated travel time *t* and S-wave velocity *V*_S_ will be incorrect. The purpose of this study is thus to delve into the effects of wave attenuation on the determination of S-wave travel time *t* and the S-wave velocity *V*_S_ in the context of the time domain method under long test distance conditions. The scope of tests conducted will be limited in partially saturated clay samples with various lengths.

## 2. Materials and Experimental Layout

### 2.1. Sample Preparation

The reconstructed silty clay was used in this study. The reason why clay is used is that it is difficult to prepare uniform sand samples with different lengths under un-confined conditions. The clay has the following Atterberg limits: plastic limit *PL* = 15% and liquid limit *LL* = 31%. The soil was first air-dried, then the soil blocks were ground and passed through a sieve to prepare the soil powder. The soil powder and water were then mixed in proportions to form a slurry. Next, the slurry was poured into a PVC tube of 60 mm in diameter and 200 mm in length, followed by a one-dimensional consolidation for 72 h under the pressure of 200 kPa. After consolidation, the soil was pushed out from the tube and maintained at room temperature for 24 h to further reduce the moisture content to about 20% and the saturation degree to 60%. Finally, a long cylindrical clay sample was trimmed out with the diameter *D* of 60 mm and the length *L* of 125 mm. This long sample was regarded as homogeneous.

### 2.2. Test Arrangement and Test Procedure

**Figure 2:**
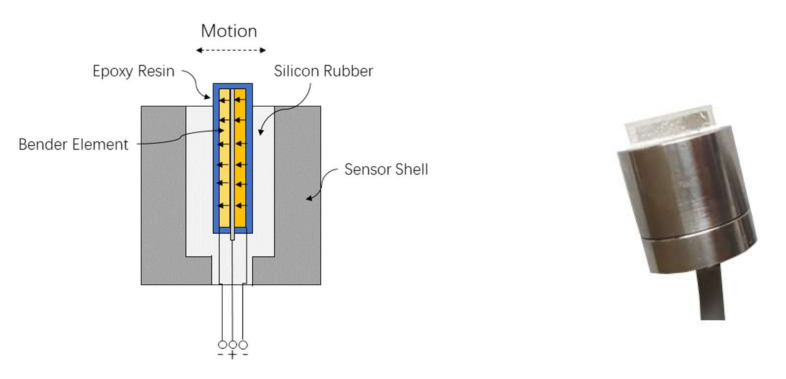
Bender element sensors and its working principle.

A pair of parallel type bender element sensors were used in this study and the structure of the sensors are shown in Figure 2. The bender element is composed of two piezoelectric bimorphs with external conducting surface, which is installed together with conductive metal in the center. Bender elements were waterproofed by an epoxy coating. The epoxy-coated bender elements are approximately 15 mm squared and 2 mm thick. It was inserted into the sensor shell. The remaining space between the shell and the bender element is filled with silicone rubber. The protruded length of bender element is 2.5 mm. The details of the connection and working principle are also shown in Figure 2. When the input voltage is applied to the transmitter, one piezoelectric sheet extends and the other contracts so that the protruded part moves in thickness direction which generates shear waves in front of the sensor and longitudinal waves on both sides, as shown in Figure 1b. In receive mode, the bender element converts the vibration into an electrical signal output. The resonant frequency of the sensor in air is determined around 3 kHz by the following method mentioned in [25]. The sensor is excited in air with an impact and corroborated by laterally pushing the bender element with a 0.5 mm lead until the lead breaks in bending to simulate a negative step excitation. Then the resonant frequency was calculated form the signal of this free vibration.

**Figure 3:**
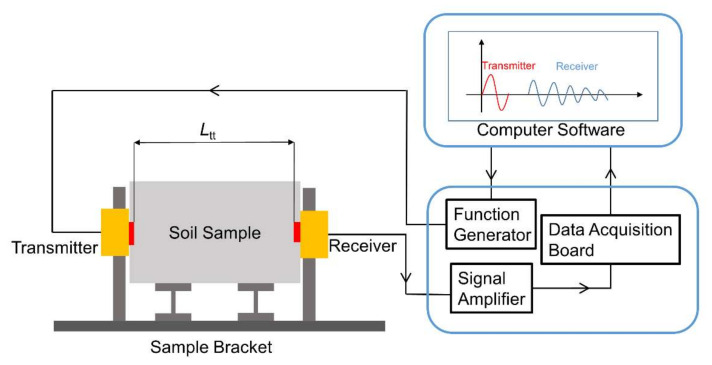
Arrangement of the bender element test.

The arrangement of the bender element test is shown in Figure 3. For continence, a sample bracket was used to fix the transmitter and receiver. The sample was fixed between the two bender elements under the unconfined conditions. The protruded part (length 2.5 mm) of the bender elements were directly inserted into the soil sample without couplant. The wave travel distance *L*_tt_ is the tip-to-tip length of the bender elements, which is 5 mm smaller than the sample length (*L*). A single cycle sinusoidal signal generated by the function generator is used as the transmitting signal. The voltage amplitude of the transmitting signal is 5 V. By changing the frequency of excitation electrical signal, the frequency of the sinusoidal transmitting signal could vary from 1 to 100 kHz. The electric signal is then converted into a shear wave propagating through the soil sample by the transmitter. Upon being received by the receiver, the shear wave movement is converted into electrical signals, which are amplified by the signal amplifier and captured by a 12-bit high-speed data acquisition board. The maximum digital storage sampling rate is 60 × 10^6^ samples per second. To improve the signal-to-noise ratio, a stacking number of 16 is used. The system delay had been calibrated to be 5 μs with the bender element tips of the transmitter and receivers in direct contact. The wave propagating through the sample bracket was calibrated to be negligible by comparing the received signals obtained under the conditions of with and without the sample bracket. In bender element testing, excitation frequencies should be properly selected in order to obtain satisfactory signals and best test results. The resonant frequency of the sensors in testing is very different from that determined in air and it is strongly influenced the properties of the soil samples [15]. In bender element test, higher excitation frequencies have the advantages of reducing the near field effect, but it may result in larger wave attenuation. Therefore, different excitation frequency is usually used in bender element test for the best test result. In the literature, the excitation frequency is mostly in the range of 2 kHz to 50 kHz [15]. In this test, input frequencies *f* selected were 5, 10, 20, and 30 kHz.

The testing process can be summarized as follows. First, the 125 mm length sample was tested. The sample was then trimmed 10 mm off and tested using the four frequencies mentioned above. This test procedure was repeated until the final sample length is 15 mm. Therefore, the number of the tested samples is 12 in total and the *L*/*D* varies from 0.25 to 2.08. After the bender element testing, the water content of each trimmed soil was measured and the corresponding values ranged from 18.45% to 18.68%. Due to the slight variation in moisture content, the sample may be considered homogeneous.

## 3. Test Results

### 3.1. Travel Time Δt and S-Wave Velocity V_S_

**Figure 4:**
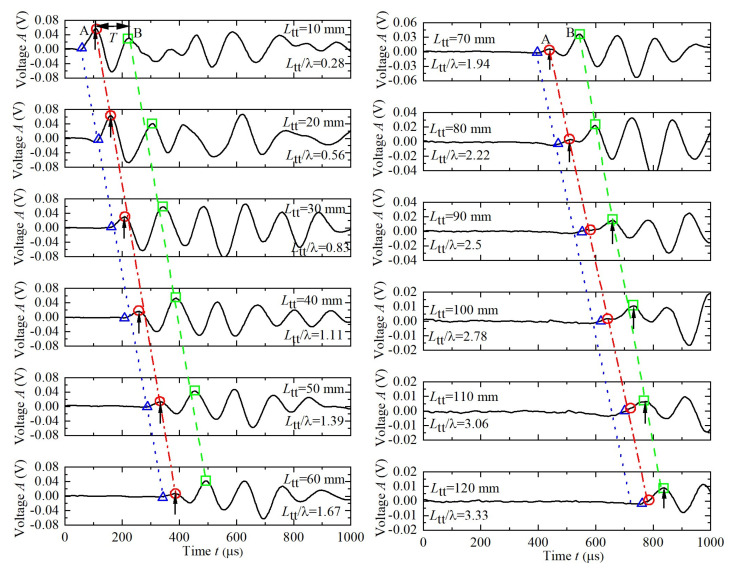
The 12 received waveforms at *f* = 5 kHz.

The received waveforms of the twelve samples measured at *f* = 5 kHz are shown in Figure 4. At this test frequency, the wave number *L*_tt_/λ are in the range of 0.28 to 3.33, in which *λ* is wave length (*λ* = *V*_S_/*f*). These waveforms depict the characteristic points including the initial arrival point (triangle), visually-identified first peaks (upward arrow), real first peaks (point A, circle) and real second peaks (point B, rectangular). The initial arrival point is determined by the zero amplitude after the first bump on received waves (i.e., the I_3_ point in Figure 1b) as proposed by [16,31]. It can be seen that there is a marked tendency for point A to attenuate gradually with the increase of *L*_tt_. Point A can be easily identified for *L*_tt_ ≤ 50 mm (*L*_tt_/λ ≤ 2.22), increasingly difficult to identify for *L*_tt_ = 60, 70, and 80 mm and fail to identify for *L*_tt_ > 80 mm. When *L*_tt_ > 80 mm, the visually-identified first peaks are in fact the second peaks, not the real first peaks.

**Figure 5:**
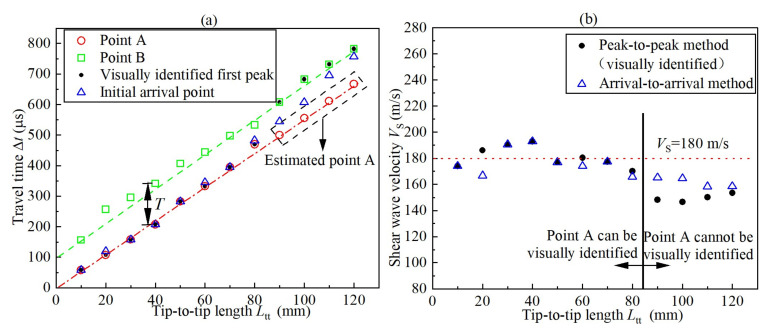
Results of (**a**) travel time Δ*t* and (**b**) S-wave velocity *V*_S_ (*f* = 5 kHz).

The Δ*t* and *V*_S_ were determined by the peak-to-peak approach using the visually-identified first peaks and the arrival-to-arrival approach using the visually-identified initial arrival points (i.e., I_3_ point) respectively. The results are shown in Figure 5a,b respectively. For *L*_tt_ ≤ 80mm, the two aforementioned approaches afford practically identical Δ*t* and *V*_S_ except for the case of *L*_tt_ = 20mm, where strong near field effect with a clear traverse waveform emerges before the S-wave arrival (see Figure 4). The *V*_S_ is distributed within a limited extent with its average value of 180 m/s. The travel time lines corresponding to *V*_S_ = 180 m/s are extended to evaluate real initial arrival points and real first peaks for *L*_tt_ > 80mm and they are shown in Figure 4 and Figure 5a with blue triangle and red circle respectively. Clearly, for *L*_tt_ > 80mm, the two approaches tend to overestimate Δ*t* and underestimate *V*_S_. Assuming the real *V*_S_ = 180m/s, the average relative errors (= (measured *V*_S_ − real *V*_S_)/real *V*_S_ × 100%) are approximately −17% and −10% for the peak-to-peak approach and arrival-to-arrival approach, respectively. In this study, arrival-to-arrival approach seems better than the peak-to-peak approach when the first peak cannot be correctly distinguished.

**Figure 6:**
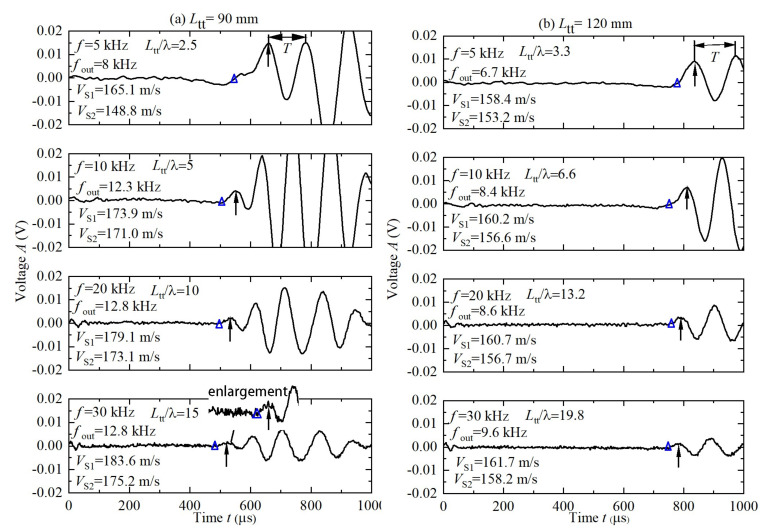
Received waveforms at four testing frequencies: (**a**) *L*_tt_ = 90 mm; (**b**) *L*_tt_ = 120 mm.

In order to investigate the influence of the test frequency on the test results, Figure 6 describes the received waveforms and the *V*_S_ determined for *L*_tt_ = 90 and 120 mm at preceding four frequencies of 5, 10, 20, 30 kHz. In Figure 6, the *V*_S1_ is the *V*_S_ determined by peak-to-peak approach and the *V*_S2_ is the *V*_S_ determined by arrival-to-arrival approach. For *L*_tt_ = 90 mm, although the real first peak was not excited when *f* = 5 kHz, it was excited when *f* ≥ 10 kHz (*L*_tt_/λ ≥ 5) and the measured *V*_S1_ and *V*_S2_ approach *V*_S_ = 180 m/s. Nonetheless, for *L*_tt_ = 120 mm, the real first peak was not excited under the four test frequencies and the measured *V*_S1_ and *V*_S2_ are approximately 155 m/s. The *f*_out_ shown in Figure 6 is the excited resonant frequency of the bender element-soil system, which was estimated with the period *T* of the received signals (see Figure 6) by *f*_out_ = 1/*T*. When test frequencies were over 10 kHz, the *f*_out_ approximately stabilized at 12 kHz for *L*_tt_ = 90 mm and 9 kHz for *L*_tt_ = 120 mm, which are larger than the resonant frequency 3 kHz obtained in air without soil sample. These results show that under the test frequency of *f* = 10 kHz which is close to the *f*_out_, the real first peak is excited when *L*_tt_ = 90 mm but not when *L*_tt_ = 120 mm. Therefore, the signal enhancement using a resonant test frequency (*f* approaching *f*_out_) proposed by some researchers [25,31,32] is efficient only within a certain test distance in this study.

### 3.2. Attenuation of Peak Points

**Figure 7:**
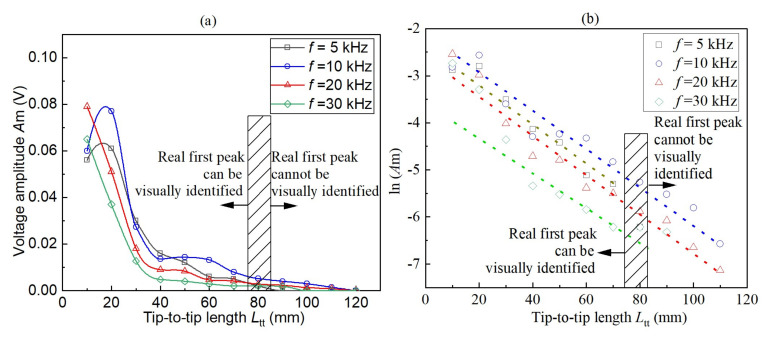
Attenuation curves of real first peak A in (**a**) *A*_m_-*L*_tt_ scale and (**b**) ln(*A*_m_)-*L*_tt_ scale.

**Figure 8:**
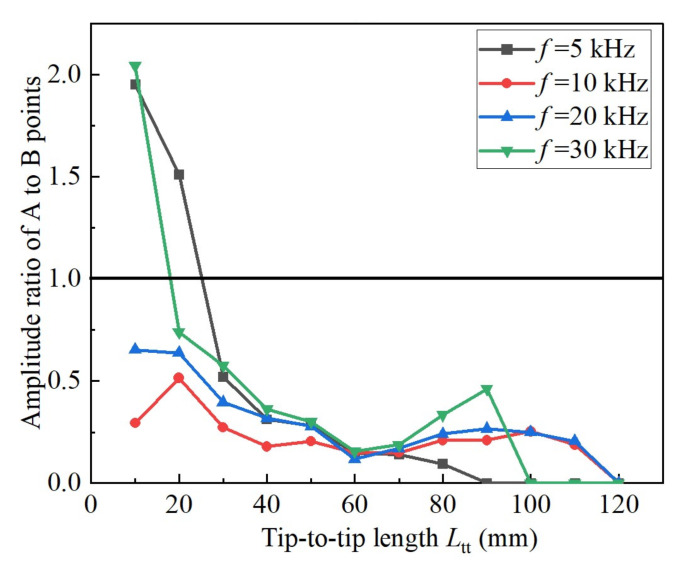
Amplitude ratio of peak point A to peak point B.

Figure 7 shows the relationships of the received voltage amplitude (*A*_m_) of the real first peak A against the test length *L*_tt_ at the four frequencies in two kinds of scales. It can be seen that *A*_m_ of real first peak A decreased significantly with the increase of *L*_tt_. The received voltage amplitude (*A*_m_) of the real first peak A is entirely below 0.08 V, which is considerably much smaller than the input voltage amplitude 5 V. This significant attenuation mainly arises from the factors including transformation between voltage and mechanical wave, bender element–soil interactions, sample absorption, and dispersion in surrounding air/materials. The critical value for the identification of first peak is approximately 0.002 V. The linear ln(*A*_m_) versus *L*_tt_ relationships shown in Figure 8 indicate the attenuation following the exponential relationship. A decreasing trend in *A*_m_ with test frequencies *f* is observed, except for *f* = 10 kHz which approaching the resonant frequency *f*_out_.

Figure 7b shows the amplitude ratio of point A to point B, i.e., real first peak to real second peak. The ratios are primarily smaller than 1.0, indicating the amplitude of point A is smaller than point B. The ratios generally decrease with *L*_tt_, indicating that point A attenuates more significantly than point B as *L*_tt_ increases. This feature is also reported in [26]. It is possibly related to the frequency components of the waveform involved: point A contains higher frequency components, thereby exhibiting more remarkable attenuation. However, this hypothesis needs to be tested with more information.

## 4. Discussion

**Figure 9:**
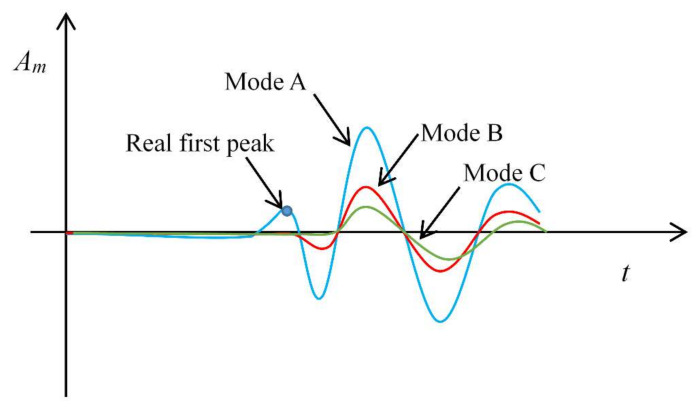
Three modes of received signals related to wave attenuation.

According to the test results, Figure 9 summarized the three modes of the received signals related to wave attenuation. Mode A is a normal one, in which the attenuation is not significant and the real first peak of the S-wave can be clearly distinguished. In mode B, wave attenuation makes the real first peak undistinguished, but the revised part of the first cycle can still be distinguished. Under this condition, the received signal is very similar to that given in Figure 1b and the revised signal is very easy to be mistaken as the P-wave distortion in near field effect or boundary reflection. In mode C, the first cycle is completely undistinguished, both peak-to-peak approach and arrival-to-arrival approach may underestimate the *V*_S_ significantly. In any case, the wave attenuation may make the over-estimation of the S-wave travel time and under-estimation of the S-wave velocity. Therefore, it is very different from the P-wave distortion mentioned in the Introduction part in this paper, which will make the over-estimation of the S-wave travel time and under-estimation of the S-wave velocity.

The errors for mode C, arising from the imperceptible first cycle of received waveform, can be estimated using the period *T* (i.e., time difference between the first peak and second peak) as follows

(2) $$\mathrm{Relative}\;\mathrm{error}\;\mathrm{of}\;V_{s}=\frac{measured\;V_{s}-real\;V_{s}}{real\;V_{s}}=\frac{\;L_{tt}/\left(\Delta t+T\right)-L_{tt}/\Delta t_{}}{L_{tt}/\Delta t_{}}=-\frac{\;T_{}}{\Delta t+T_{}}\left(\times 100\%\right)$$

in which Δ*t* is real travel time and Δ*t+T* is visually-identified travel time with an error of *T* because of attenuation. According to Figure 5a, the period *T* maintains almost constant at 100 μs under *f* = 5 kHz for various *L*_tt_. According to Equation (2), because Δ*t* increases with *L*_tt_, the relative error will decrease with *L*_tt_. It agrees well with results of peak-to-peak approach given in Figure 5b. The deviation of arrival-to-arrival approach from Equation (1) is presumably attributed to the complexity of waveform around the S-wave arrival: a slight reverse occurs before the arrival of S-wave when *L*_tt_ ≥ 80 mm (see Figure 4). This slight reverse is possibly resulting from the light boundary reflection of P wave under long test distance [24,33].

Ingale et al. (2017) summarized the factors influencing the wave attenuation in bender element tests [15]. The absorbing attenuation is closely associated with the damping characteristics of soil which is determined by soil type, moisture content, and stress state [17,34,35]. The influence of testing confining pressure on the received waveform has been studied with contrary conclusions. For example, the results of [36] using fully saturated kaolin in ultrasonic testing show that wave attenuation can be reduced with an increase of confining pressure. Conversely, other researchers observed that the amplitude of the received waveform decreases with increasing confining pressure in their bender element tests using dry sand [33,37]. Arguably, the consolidation process and confining pressure may be beneficial to lessen the wave attenuation effect merely for clay samples. Adopting higher length-to-thickness ratio to achieve low resonant frequency is also an available method to reduce the wave attenuation [15]. In principle, wave attenuation can also be reduced by improving the performance of testing system. Approaches include: (1) using the digital oscilloscopes with high analog to digital conversion resolution (≥12 bits) [21,38], (2) parallel connection and series connection as a transmitter and a receiver, respectively [21], (3) increases the signal to noise ratio of the received waveforms by using a large voltage applied to transmitters [21]. However, theoretically the wave attenuation effect will always matter when *L*_tt_ exceeds a critical value, which is concerned with material properties, test equipment, sample characteristics, and test conditions. Consequently, it is advisable to carefully pre-determine the corresponding critical length of the test system using different sample length or test distance, so that the accurate velocity can be obtained.

## 5. Conclusions

Wave attenuation in bender element testing has been observed here to serve as a contributing factor influencing received waveforms and shear wave velocity determination by the time domain method for long samples. In this study on unsaturated clay samples, the wave arrival time is prone to be misinterpreted for the cases with wave travel distance over approximately 80 mm, thereby leading to underestimation of the *V*_S_ by 17% on average for the peak-to-peak approach and 10% and on average for the arrival-to-arrival approach. Three modes of the received signals related to wave attenuation are summarized. In any case, the wave attenuation may make the over-estimation of the S-wave travel time and under-estimation of the *V*_S_. This effect is fundamentally different from the P-wave distortion, which results in the over-estimation of the S-wave travel time and under-estimation of the *V*_S_. The wave attenuation effect may be minimized with an appropriate excitation frequency or improved testing system. However, given that this effect cannot be totally eliminated in theory, when involving long distance testing with high test frequency, it is suggested that the wave attenuation characteristic and critical test length for a testing system should be calibrated with caution.

## Figures and Tables

**Table 1:** Sample length and test devices/conditions in some selected studies.

References	Test Devices/Conditions	Material	Sample Dimensions or Test Distance (mm)	
			Length (*L*)	Diameter (*D*)
[26]	Triaxial test	Consolidated clay	22–95.8	≈50
[20]	Model box	Toyoura sand	60–160	/
[27]	Oedometer test	Gault clay	70–150	90
[28]	Resonant column test	Toyoura sand	100	50
[11]	Unconfined	Soft clays mixed with ordinary Portland cement	100	50
[29]	Confined by tube	Dry sand	120–320	50
[30]	Triaxial test	Residual soil from Porto granite	140	70
[9]	Triaxial test	Clay	150	75
[18]	Triaxial test	Fibrous peat organic soil	154	72

## Data Availability

The data presented in this study are available on request from the corresponding author.
